# Validity of a Protocol for Adult Self-Report of Dyslexia and Related Difficulties

**DOI:** 10.1002/dys.1432

**Published:** 2012-02

**Authors:** Margaret Snowling, Piers Dawes, Hannah Nash, Charles Hulme

**Affiliations:** 1 University of York - PsychologyYork, UK; 2 University of Manchester - Audiological SciencesManchester, UK; 3 University of York - PsychologyYork, UK; 4 Division of Psychology and Language Sciences, University College LondonUK

**Keywords:** dyslexia, adults, attention, screening, diagnosis

## Abstract

**Background:**

There is an increased prevalence of reading and related difficulties in children of dyslexic parents. In order to understand the causes of these difficulties, it is important to quantify the risk factors passed from parents to their offspring.

**Method:**

417 adults completed a protocol comprising a 15-item questionnaire rating reading and related skills and a scale assessing ADHD symptoms; 344 completed reading, nonword reading and spelling tests.

**Results:**

A confirmatory factor analysis with four factors (Reading, Word Finding, Attention and Hyperactivity) provided a reasonable fit to the data. The Reading Factor showed robust correlations with measured literacy skills. Adults who reported as dyslexic, or rated their reading difficulties as more severe, gained lower scores on objective measures of literacy skills. Although the sensitivity of the new scale was acceptable, it tended to miss some cases of low literacy.

**Conclusions:**

Self-report scales of reading and of attention difficulties are useful for identifying adults with reading and attention difficulties which may confer risks on their children of related problems. It is important for research following children at family risk of dyslexia to be aware of these effects. Copyright © 2012 John Wiley & Sons, Ltd.

Dyslexia is a neurodevelopmental disorder which primarily affects the development of reading accuracy, fluency and spelling skills (IDA, 2002; American Psychiatric Association, 2011). For some individuals with dyslexia, reading difficulties may be overcome leaving impairments only in spelling and aspects of phonological processing (Bruck, 1992; Ramus *et al.*, 2003). For others, dyslexia persists well beyond the school years and may affect adult career and employment prospects (Maughan *et al.*, 2009).

One obstacle to the identification of dyslexia is a lack of consensus concerning its defining symptoms, and debate concerning the core characteristics of dyslexia has continued for more than 40 years (Snowling, 2009; Vellutino, Fletcher, Snowling & Scanlon, 2004 for reviews). Increasingly, it is accepted that dyslexia is not an ‘all or none’ condition but rather a dimensional disorder underpinned by poor phonological skills (Pennington & Lefly, 2001; Hulme & Snowling, 2009). In addition, there is recognition that dyslexia tends to co-occur with other language and learning disorders (Pennington & Bishop, 2009). An individual is more likely to receive a ‘diagnosis’ of dyslexia if they have relatively severe reading and spelling difficulties or if their literacy difficulties are compounded by co-morbid difficulties with language or attention (Snowling, 2008). Indeed, problems of language and of attention are commonly considered to be dyslexia-associated traits (Rose, 2009).

In recent years, there has been a growth of interest in children at family risk (FR) of dyslexia across languages (e.g., in English, Scarborough, 1990; in Finnish, Lyytinen *et al.*, 2006; in Chinese, McBride-Chang *et al.*, 2011). An important aim of these studies is to identify the precursors of dyslexia before reading instruction begins. Equally important is to understand the inter-generational transfer of risk between parents and their offspring. Thus, van Bergen, de Jong, Plakas, Maasen and van der Leij (2012) showed that parental reading skills contributed unique variance to the prediction of children's reading fluency even when child-level predictors, such as phonological awareness, were controlled.

To date, the majority of studies of children at FR have screened parents only for literacy and related phonological skills (e.g., Snowling, Gallagher & Frith, 2003) or, in some cases, have relied on self-report of dyslexia (e.g., Hindson *et al.*, 2005). Since current theories suggest dyslexia is the behavioural outcome of multiple risks acting together to increase the probability of poor reading (Pennington, 2006), it follows that family-risk studies need to incorporate procedures for estimating risk not only of dyslexia but also of commonly co-occurring disorders. Such disorders include Attention Deficit Hyperactivity Disorder, a disorder involving difficulties with the control of attention and behaviour associated with symptoms of inattention, hyperactivity/impulsivity or both (e.g., McGrath *et al.*, 2011) and Language Impairment, a disorder characterized by delayed language development alongside normal nonverbal ability (Pennington & Bishop, 2009); both of these disorders, like dyslexia, are dimensional in nature and vary in severity.

Questionnaires and rating scales provide a time-saving way of estimating risk factors for dyslexia. Many dyslexia screening questionnaires include not only questions about literacy skills but also items which tap constructs, such as problems of attention, organization and word finding (e.g., Cooper & Miles, 2011; Smythe & Everatt, 2001; Vinegard, 1994). Potentially, these questionnaires offer additional information relevant to the quantification of risk, but few are validated. The present study investigated whether a self-report questionnaire could provide a valid measure of literacy, and further, whether this measure could be useful as part of a protocol for the identification of ‘dyslexia’ and co-occurring problems in adulthood. The primary reason for validating the protocol was for use in family studies investigating risk factors for disorders. We describe data collected from parents of pre-school children. The study sample as a whole was diverse in terms of educational level, socio-economic background and occupational status and included people with dyslexia and was therefore considered appropriate for the validation of a dyslexia-risk screening protocol.

A number of previous studies have assessed the validity of questionnaires and interviews for the self-report of reading difficulties (Gilger 1992; Gilger, Pennington & DeFries, 1991; Pennington & Lefly, 2001; Schulte-Körne, Deimel & Remschmidt, 1997). Evidence of the predictive validity of self-report comes from its relationship to measured reading and spelling skills as well as to the emergent reading skills of the offspring of respondents (Elbro, Nielsen & Petersen, 1994). Wolff and Lundberg (2003) found that a self-report measure of dyslexia correlated well with measures of word recognition in a group screening of Swedish adults. Lindgren and Laine (2007) also provided validation data from a Finnish version of the same measure. However, Gilger (1992) showed that the accuracy of retrospective self-report of academic attainments in school depends upon a number of factors including age, gender, level of achievement and history of learning disabilities. How to interpret these effects is unclear. We first sought to replicate and extend the findings to the self-report of dyslexia and then to gain better understanding of the weaknesses of self-report methods by considering the extent to which they under- and over-identify literacy difficulties.

In summary, in the present study, we set out to validate a protocol to assess dyslexia and dyslexia-associated traits in adults. We anticipated that self-reported dyslexia should differentiate people with and without dyslexia-associated traits, namely problems of word finding, organization, attention and hyperactivity. Also, we expected that people who rated their reading difficulties/dyslexia as more severe should gain lower scores on measures of literacy. Finally, given that reading difficulties tend to be associated with low levels of print exposure both in children and in adults (Cunningham & Stanovich, 1997; Maughan *et al.*, 2009, Snowling, Muter & Carroll, 2007), we expected that ratings of reading difficulty would predict a lower frequency of literacy-related activities in everyday life.

## Method

### Participants

#### Recruitment

Beginning in October 2007, families with a history of dyslexia with 3-year-old children (FR) and similar families with no history of reading difficulties (typically developing (TD)) were recruited to the ‘Wellcome Language and Reading Study’ via advertisements placed in local newspapers and on the webpages of local and national support agencies for children with reading difficulties/dyslexia and language difficulties. In addition, the families of children with language difficulties (LI) were referred by speech and language therapy services and nurseries or recruited at drop-in clinics for parents with concerns about their child's speech/language development. Ethical permission for the study was granted by the University of York, Department of Psychology's Ethics Committee and the NHS Research Ethics Committee. All families involved in the study provided informed consent.

#### Study Sample

At the time of the present analyses, 417 parents of children participating in the ‘Wellcome Language and Reading Study’ completed the Adult Reading Questionnaire (ARQ): 170 were from the FR group, 91 were parents of children in the language impaired (LI) group and 156 were from families with no known or suspected difficulties (TD group). It should be noted that the classification of parents was according to the status of their offspring and is not of prime concern here.

The mean age of the parent sample was 36.17 years, SD = 6.33. The sample comprised 238 mothers and 179 fathers. Of these, 344/417 (82%) agreed to be assessed on psychometric tests. Eighty four parents reported that they were ‘dyslexic’, but only 31 of these had received a formal diagnosis.

## Design and Materials

Each parent of a child in the study was asked to complete the new ARQ, the Adult ADHD Self-Report Scale (ASRS, a 6-item screening measure for ADHD in adults, Kessler *et al.*, 2005) and two further questionnaires not reported here, one assessing language and communication skills and one assessing general health. Parents who gave consent also completed a battery of psychometric tests tapping IQ, literacy and language skills. For the purposes of the present validation study, data are analysed from the ARQ (see below), the ASRS and from tests of reading and spelling. In addition, information about the parents' educational level and occupation was gathered during a structured interview.

### Psychometric Test Battery

#### Nonverbal Ability

The Wechsler Abbreviated Scale of Intelligence (WASI, Wechsler, 1999) Block Design subtest was given to provide an assessment of nonverbal IQ. In this test, the participant has to assemble sets of blocks to match a series of 2-D patterns presented in a booklet. Time and accuracy were recorded as specified in the Test Manual.

#### Vocabulary

The WASI (Wechsler, 1999) Vocabulary subtest was given as a proxy for verbal IQ. In this test, the participant has to define a series of words of increasing difficulty. Answers are scored for depth of vocabulary knowledge as specified in the Test Manual.

#### Reading Skills

The Test of Word Reading Efficiency (Torgesen, Wagner & Rashotte, 1999) was given to assess participant's ability to pronounce printed words accurately and fluently. The test comprises tasks tapping word (Sight Word Efficiency) and nonword reading efficiency (Phonemic Decoding). The test was scored according to the manual.

#### Spelling Skills

Participants completed the Spelling Subtest from the Wide Range Achievement Test (WRAT 4; Wilkinson & Robertson, 2006). The test was scored according to the manual.

### Questionnaires

The screening protocol was devised to tap poor reading and writing skills as well as expressive language difficulties (word finding) since these symptoms often occur together in people with dyslexia and are frequently included in dyslexia assessment schedules (e.g., the Bangor Dyslexia Test, Miles, 1997). In addition, the protocol aimed to screen for problems of organization, attention and hyperactivity which are sufficiently common to be regarded as dyslexia-associated traits.

#### Adult Reading Questionnaire

Items to assess aspects of literacy, language and organization were drawn from a number of sources, including the Adult Dyslexia Checklist (Smythe & Everatt, 2001). The new scale comprised 15 items: 7 items required the respondent to rate ‘symptoms’ of dyslexia on a scale of 0–4, such as problems with literacy skills, word finding and organization (e.g., *Do you find it difficult to read words you haven't seen before*? Never / Rarely / Sometimes / Frequently / Always), 2 further items required a yes/no/maybe response (e.g., *Are you a good reader*?) and 2 items required the respondent to rate how frequently they read and write (e.g., *How often do you write in everyday life*?). In addition, the questionnaire contained the following definition:

#### Dyslexia is difficulty with reading and writing in people who

- do OK in other aspects of life (their difficulty is mostly with reading and writing)
- have had the chance to learn to read, but have not been able to learn like others

Following this, 4 items specifically asked about a dyslexia diagnosis:

*Based on this, do you think you are dyslexic?* (yes/no/maybe)
*How would you rate your difficulties?* (no difficulties/ mild / moderate/ severe)
*Has anyone ever raised concerns about your reading?* (yes/no)
*Have you ever had a diagnosis of dyslexia?* (yes/no) *If YES, by whom?*

Items from the questionnaires were scored numerically, with higher scores associated with more severe difficulty or greater likelihood of impairment. Item scores ranged from 0–1 or 0–4, depending on the question (see Appendix A for ARQ items and scoring details).

#### Adult ADHD Self-Report Scale

The World Health Organization Adult ADHD self-report scale (ASRS; Kessler *et al.*, 2005) was used to assess symptoms of attention deficit and hyperactivity which are frequently co-morbid with dyslexia. The scale comprises 6 items, 4 tapping difficulties with sustained attention, organization and prospective memory, and 2 tapping hyperactivity.

## Procedure

The questionnaires and psychometric tests were completed by parents when their child was first seen at 3;06 years approximately, either while their child was being assessed (by a second tester) or during a separate visit, particularly in the case of working parents. Questionnaire items were given in interview format in a small number of cases when a parent indicated this was their preference and the examiner recorded responses.

## Results

Descriptive statistics for responses to the ARQ from 417 adults, excluding their responses to the 4 dyslexia questions, are shown in the upper rows of Table 1, with the responses to the items of the ASRS in the lower 6 rows. It is clear that each of the questions yielded a good range of scores (from the minimum to the maximum in each case). We began by examining the intercorrelations among items from the two scales before assessing the factorial structure of the protocol. Four factors emerged tapping Reading, Word Finding, Attention and Hyperactivity. We proceeded to validate the Reading factor scores against measures of literacy before comparing adults who self-reported as dyslexic on the four constructs (there was a good spread of literacy scores in the sample). Finally, using an iterative procedure, we estimated the cut-off scores which would provide a sensitive assessment for literacy difficulties.

**Table 1:** Descriptive statistics for each of the items on the Adult Reading Questionnaire and ASRS

	Mean	SD	Min	Max
ARQ1 Do you think you are a good reader?	0.172	0.36	0	1
ARQ2 Can you read quickly and easily?	0.21	0.4	0	1
ARQ3 How good is your spelling?	0.84	0.81	0	3
ARQ4 In your job, how often do you read?	0.91	0.95	0	4
ARQ5 Do you find it difficult to read words you haven't seen before?	1.48	1	0	4
ARQ6 Do you find it difficult to read aloud?	1.19	1.15	0	4
ARQ7 Do you find it difficult to find the right word to say?	1.59	0.81	0	4
ARQ8 Do you ever confuse the names of things?	1.29	0.88	0	4
ARQ9 Do you confuse left and right?	0.96	1.18	0	4
ARQ10 Do you have problems with organization or time management?	1.3	1.04	0	4
ARQ11 How often do you write in everyday life?	1.03	0.95	0	4
ASRS1 How often do you have trouble wrapping up the fine details of a project, once the challenging parts have been done?	1.57	0.99	0	4
ASRS2 How often do you have difficulty getting things in order when you have to do a task that requires organization?	1.26	0.87	0	4
ASRS3 How often do you have problems remembering appointments or obligations?	1.46	1	0	4
ASRS4 When you have a task that requires a lot of thought, how often do you avoid or delay getting started?	1.86	0.92	0	4
ASRS5 How often do you fidget or squirm with your hands or feet when you have to sit down for a long time?	1.83	1.16	0	4
ASRS6 How often do you feel overly active and compelled to do things, like you were driven by a motor?	1.66	1.09	0	4

### 

#### Factor Structure of the Protocol

We first computed correlations between each of the items in the ARQ and the ASRS. We omitted the four dyslexia questions and the two questions assessing frequency of reading and writing (ARQ4 ‘*How often do you read in your job*’; ARQ11 ‘*how often do you write in everyday life*’) from this analysis because we wished to use these later for the purpose of validation.

Table 2 shows the correlations between items. It can be seen that the questions tapping the four putative constructs, Reading, Word Finding, Attention and Hyperactivity tend to inter-correlate better than items tapping different constructs: Group 1 comprises ratings of reading (and spelling) skills (ARQ1, ARQ2, ARQ3, ARQ5, ARQ6); Group 2 comprises ratings of word finding/labelling skills (ARQ7, ARQ8, ARQ9); Group 3 comprises ratings of attentional/organizational skills (ARQ10, ASRS1, ASRS2, ASRS3, ASRS4); Group 4 comprises only the two items tapping hyperactivity ASRS5, ASRS6). It is clear that one pair of questions measuring reading (ARQ1 and ARQ2) have a very high correlation with each other (*r* = .81); essentially, these questions amount to almost alternate forms of the same question (‘*Do you think you are a good reader*’ and ‘*Can you read easily and quickly*’). One of the questions from the ARQ (*Do you have problems with organization or time management*?) correlated moderately with items tapping attention from the ASRS but otherwise the intercorrelations between the two scales were low.

**Table 2:** Intercorrelations of test items from the Adult Reading Questionnaire and the ASRS

	ARQ1													
ARQ2	0.81	ARQ2												
ARQ3	0.54	0.51	ARQ3											
ARQ5	0.50	0.50	0.59	ARQ5										
ARQ6	0.62	0.61	0.52	0.58	ARQ6									
ARQ7	0.26	0.30	0.32	0.39	0.38	ARQ7								
ARQ8	0.24	0.32	0.30	0.41	0.35	0.51	ARQ8							
ARQ9	0.14	0.16	0.25	0.28	0.28	0.22	0.36	ARQ9						
ARQ10	0.12	0.15	0.10	0.15	0.18	0.29	0.31	0.32	ARQ10					
ASRS1	0.22	0.19	0.25	0.30	0.24	0.36	0.35	0.26	0.50	ASRS1				
ASRS2	0.24	0.23	0.22	0.32	0.29	0.37	0.36	0.30	0.35	0.59	ASRS2			
ASRS3	0.18	0.18	0.19	0.13	0. 11	0.25	0.20	0.37	0.17	0. 41	0.44	ASRS3		
ASRS4	0.20	0.16	0.15	0.24	0.23	0.21	0.24	0.22	0.43	0.47	0.54	0.35	ASRS4	
ASRS5	0.16	0.15	0.20	0.20	0.23	0.30	0.25	0.13	0.22	0.28	0.31	0.28	0.28	ASRS5
ASRS6	0.16	0.15	0.20	0.15	0.15	0.19	0.19	0.13	0.0	0.19	0.18	0. 13	0.11	0.42

**Figure 1:**
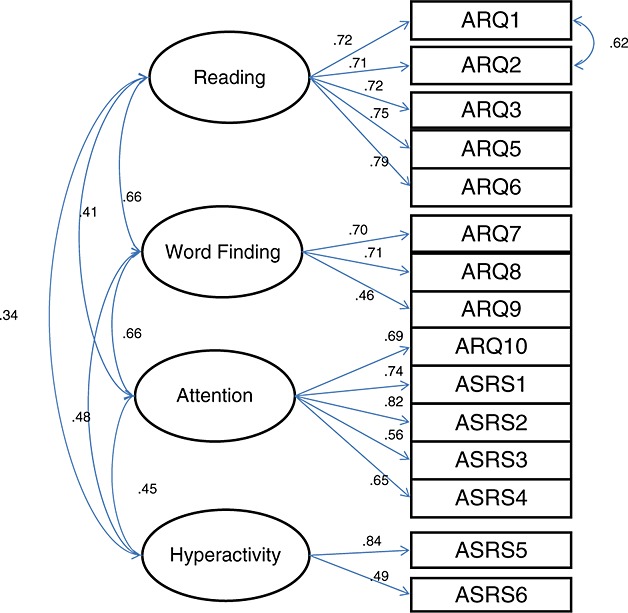
Factor structure of the ARQ.

Based on the theory guiding the construction of the reading questionnaire with items tapping literacy, word finding and organization, and taking account of the pattern of correlations between the items and those of the ASRS, we conducted a Confirmatory Factor Analysis to assess the fit of the data to four factors (Reading, Word finding, Attention and Hyperactivity). The analysis was conducted in MPlus v6.0, with maximum likelihood estimation. The small proportion of missing data (less than 1%) was handled using Full Information Maximum Likelihoods (the default in MPlus). The model also allowed correlated errors of measurement between ARQ1 and ARQQ2. This is reasonable, since as noted already, the correlation between these two questions is very high, and higher than the other questions used to define the Reading latent variable. The resulting model is shown in Figure 1. The model provides a fairly good fit to the data (χ^2^ df 83 =163.48, *p* < .001; CFI = 0.966; RMSEA = 0.048 (90% CI = 0.037–0.059)), and is significantly better than a model without correlated errors between Q1 and Q2, (χ^2^ diff (114.94, df 1, *p* > .001)).

As can be seen from the Figure, the four latent factors correlate moderately. Three of the factors have good reliability (Reading, alpha = 0.81, Word Finding, alpha = 0.60; Attention, alpha = 0.81. The reliability of the Hyperactivity factor (which only has 2 items) is modest, alpha = 0.58. For each factor, we computed factor scale scores by summing the scores on the questions that had been identified as defining each factor (Pedhazur & Schmelkin, 1991) rather than using the resulting factor scores themselves.

#### Validation of the Adult Reading Questionnaire

The four-factor structure of the protocol was the starting point for assessing the validity of the new ARQ. If the Reading Scale is a valid measure, then Reading Scale scores should correlate highly with measures of reading and spelling and moderately with verbal IQ. In contrast, lower correlations would be expected with the Word Finding, Attention and Hyperactivity Scale scores.

#### Construct Validity

We began by examining the predictive validity of the four factors in relation to measures of literacy. We expected the Reading Scale score but not the other scale scores to correlate strongly with measured reading and spelling skills. The maximum score for each scale was: Reading Scale = 13, Word Finding Scale =12; Attention Scale = 20, Hyperactivity Scale = 8.

Table 3 shows the correlations between the Scale scores and raw scores on measures of word and nonword reading, spelling, block design and vocabulary tests. Given the large sample size, all coefficients above .10 are statistically significant, and it is the pattern of correlations which is important. Scores for the Reading Scale correlated highly with measures of nonword reading (−.66) and spelling (−.60); the correlation with word reading was moderate (−.51) suggesting that word reading fluency is not as sensitive a measure as spelling and nonword reading fluency in this population. We also derived a composite measure of literacy skill from the two measures most likely to differentiate dyslexic from non-dyslexic reading by summing standardized scores for nonword reading and spelling). This literacy composite score correlated with the Reading Scale (*r* = −.67). In contrast, correlations between the Word Finding, Attention and Hyperactivity Scale scores and literacy measures were low. Correlations between the Scale scores and measures of verbal and nonverbal ability were low to negligible.

**Table 3:** Correlations of ARQ Scale scores and measures of reading, spelling and general cognitive ability

	Reading Scale	Word Finding Scale	Attention Scale	Hyperactivity scale
Word Reading	−0.51	−0.30	−0.14	−0.07
Nonword Reading	−0.66	−0.33	−0.15	−0.18
Spelling	−0.60	−0.28	−0.09	−0.17
Vocabulary	−0.22	−0.17	0.02	−0.14
Block Design	−0.09	−0.14	0.04	0.02

We next compared the Scale scores of the individuals who self-reported as dyslexic according to the response to the question ‘*Do you think you are dyslexic*?’ (*N* = 86) with those who did not (*N* = 331). The responses ‘Yes’ and ‘Maybe’ were coded as indicative of dyslexia. The data clearly demonstrate that parents who reported themselves as dyslexic (self-reported dyslexic; SRD) rated themselves more highly on the Reading scale than those who did not (NoSR): (NoSR Mean = 2.82 (CI 2.60-3.04); SRD Mean = 8.11 (CI 7.54-8.69)). It is noteworthy that this was also the case for self-ratings of Word Finding (NoSR Mean = 3.37 (CI 3.17-3.58); SRD Mean = 5.61 (CI 5.09-6.03)), Attention (NoSR Mean = 6.93 (CI 6.57-7.29); SRD Mean = 9.47 (CI 8.59-10.36)) and Hyperactivity (NoSR Mean = 3.21 (CI 3.02-3.41); SRD Mean = 4.5 (CI 4.10- 4.90)), providing validation for the selection of dyslexia-associated traits. Second, as shown in Table 4, Rated Severity of reading problems (the response to question ARQ19, *How would you rate your difficulties*?) correlated positively with the Reading Scale score and negatively with measures of reading and spelling.

**Table 4:** Correlations between frequency of reading and writing, how severely individuals rate their dyslexic difficulties, Reading Scale score and measures of reading, nonword reading and spelling

	Frequency Read (ARQ4)	Frequency Write (ARQ11)	Rated Severity (ARQ19)	Reading Scale	Word Reading	Nonword Reading
Frequency Write	0.55					
Rated Severity	0.19	0.26				
Reading Scale	0.23	0.27	0.72			
Word Reading	−0.20	−0.23	−0.54	−0.59		
Nonword Reading	−0.18	−0.17	−0.68	−0.72	0.74	
Spelling	−0.21	−0.20	−0.63	−0.68	0.62	0.79

Finally, we tested the prediction that ratings of reading difficulty would predict a lower frequency of literacy-related activities in everyday life by examining correlations between the questions asking about the frequency of reading and writing in everyday life (ARQ4, ARQ11) and Rated Severity of dyslexia impairment (ARQ19). Contrary to prediction, as shown in Table 4, ratings of the Frequency of Reading and Writing correlated only weakly with scores on the Reading scale and measures of reading and spelling.

#### Discriminant validity

If the ARQ is to be used as a questionnaire for the self-report of reading difficulties, then it is important to assess its ability to discriminate between those with and without reading problems at the individual level. The sample of parents who took part in this study were self-selecting, and the mean literacy level for the group whose children were TD was above average. In order to set a criterion against which to define an individual as having low literacy, it was important to take account of this recruitment bias. Since poor nonword reading and spelling are typically considered markers of ‘dyslexia’, the criterion was set in relation to the composite literacy measure of nonword reading and spelling described above. The mean for the parents of the TD children on this composite measure was 107.54, (SD =10.57; range = 71.5 -132.5). The criterion for ‘poor literacy’ (a putative marker of dyslexia) was taken to be a composite score of below 90 (approximately 1.5 SD below the TD mean): 78/343 individuals fulfilled this criterion (one participant who was assessed had missing data on these two tests).

A series of logistic regression analyses were conducted to assess how well scores on the ARQ could be used to classify individuals according to measured literacy status (Poor versus Normal). Individual scores on the Reading Scale successfully predicted literacy status, (χ^2^ (1) = 107.08, p < .001, OR = 1.63). However, although the specificity of this means of classification was high (95.85%), suggesting that low scores on the Scale are unlikely to be obtained by poor readers, its sensitivity was low (47.44%), indicating that it misses many positive cases of poor literacy. It is interesting in this light to compare the success of the dyslexia question ‘*Do you think you are dyslexic?*’ for making this classification. When responses to this question were dichotomized and used to predict literacy status, the model was significant (χ^2^ (1) = 109.24, p < .001, OR = 27.11); in this case, sensitivity was acceptable (66.67%), and specificity remained high (93.13%). Finally, we examined the adequacy of a model in which both the Reading Scale score and the Dyslexia Status question were used to predict literacy status (data were available from 334 parents on all 3 variables). This model was significant (*χ^2^* (2) = 139.50, *p* < .001) as were both predictors (Reading Scale, OR = 1.51; Dyslexia Status, OR = 5.02); sensitivity was acceptable (62.5%) and specificity was high (95.04%). This latter model gave the highest correct classification of literacy status of the three models tested (88.02%) (see Table 5).

**Table 5:** Classification of participants according to literacy status using Reading Scale and Dyslexia Self-Report Question Scores

Classified	Poor Literacy	Literacy Normal	
Poor Reader [+]	45	13	58
Normal Reader [−]	27	249	276
Total	72	262	334

Finally, from the perspective of family-risk studies, it is important to know about factors which may affect an individual's tendency to self-report as dyslexic. While it is fair to assume that literacy level will itself be an important predictor, Gilger (1992) found that age, gender, social class and educational level played a significant role. Table 6 shows the results of two regression models assessing the role of literacy skill, age, gender, educational level and social class as predictors of self-report of dyslexia.

**Table 6:** Hierarchical regression models predicting tendency to self-report as dyslexic from age, gender, educational level and social class

		Dyslexia Status			
			
		Coefficient	*t*	*p*	Total R^2^
1	Literacy	−0.20	−13.69	0.00	
2	Age	0.01	2.31	0.02	
	Gender	0.08	2.20	0.03	
	Educational Level	0.03	1.78	0.08	
	Social Class	0.00	−0.19	0.85	.41
2	Age	0.01	2.14	0.03	
	Gender	0.08	2.16	0.03	
	Educational Level	0.03	2.07	0.04	.42

Measured literacy skills accounted for 39% of the variance in the prediction of dyslexia self-report; after literacy skills were controlled, age and gender but neither educational level nor social class continued to make a significant contribution (an additional 2%) to self-report status. However, there was substantial collinearity between educational level and social class; when social class was dropped from the model, educational level was a significant predictor. In summary, the tendency to self-report as ‘dyslexic’ was predicted by literacy skills, but over and above these, more older parents, more fathers and more individuals of higher educational level tended to self-report.

## DISCUSSION

The main aim of the present study was to develop and evaluate a protocol for identifying dyslexia and related difficulties in adults. A specific objective was to design a self-report scale that could be used in family-risk studies to provide a means of estimating the multiple risks associated with dyslexia that a parent confers on their child. Hence, it was important to include items that assessed domains beyond literacy per se and included measures of attention control and expressive language, specifically word finding. The present protocol included a new ARQ asking for ratings of reading and spelling proficiency, frequency of reading and writing on an everyday basis and self-report of dyslexia, as well as completion of a validated screening tool for attention deficit hyperactivity disorder. We assessed the factor structure of the protocol and the validity of the constructs it measured in relation to behavioural measures of reading and spelling.

A four-factor measurement model (Reading, Word Finding, Attention and Hyperactivity) provided a reasonably good fit to the data from the two questionnaires. The Reading and Word Finding factors correlated strongly, as did the Word Finding and Attention Factors, with the remaining intercorrelations being moderate. The Reading, Word Finding and Attention factors showed reasonable reliability, the Hyperactivity Factor (defined by two items) was less reliable.

The validity of the new reading questionnaire (ARQ) appears to be good. Importantly, the Reading Factor showed strong concurrent relationships with measured literacy skills (particularly decoding fluency and spelling). Furthermore, adults who self-reported as ‘dyslexic’ gained lower scores on the Reading Scale than those who did not. In addition, the Rated Severity of individuals' reading difficulties correlated with their scores on the Reading Scale and, consistent with previous research, negatively with measures of reading and spelling (Gilger 1992; Gilger *et al.*, 1991; Schulte-Körne *et al.*, 1997; Wolff & Lundberg, 2003). However, contrary to prediction, Severity of difficulties was only weakly related to Frequency of Reading and Writing in everyday life.

Together these findings indicate that the ARQ provides a valid continuous measure of literacy skills, and, if used together with the ADHD screener, the protocol can identify dyslexia-associated traits including difficulties with expressive language (word finding) and attention. The protocol may therefore be useful as a way of estimating some of the risk factors (or endophenotypes) involved in the etiology of dyslexia in ‘at-risk’ children. Furthermore, the current findings fit well with causal hypotheses of dyslexia. First, it has been suggested that the learning to read capitalizes on the neural circuitry involved in object naming (Price & McCrory, 2005, Lervåg & Hulme, 2009). Consistent with this view, there was a high correlation between Reading and Word finding Scales. Second, the correlation of Attention and Word Finding suggests that a frontal brain network is associated with both word retrieval and attention control, as might be predicted from the high degree of association between dyslexia and symptoms of inattention (Carroll, Maughan, Goodman & Meltzer, 2005).

However, it is important to signal a note of caution about using a self-report measure for the purposes of classifying individuals. While there were robust correlations between the Reading Scale scores and behavioural measures of literacy, this study identified a substantial number of people with low levels of literacy who did not rate themselves as having poor reading and spelling skills or who did not self-report as dyslexic. The reasons for this under-reporting are likely to be diverse as also noted by Gilger (1992). Indeed, over and above objective measures of literacy skill, there was a stronger tendency for fathers, for older parents and for those with higher educational qualifications to self-report as dyslexic. It is difficult without more data to understand an individual's propensity to self-report dyslexic symptoms. Speculatively, and based on anecdotal evidence, it may be that it is socially more acceptable for men to admit dyslexia, and they may often be encouraged by their partners to do so. Theoretically, however, there is growing evidence that people rate their own emotional state in relation to their social group (e.g., happiness; Boyce, Brown & Moore, 2010). Applying this hypothesis to the current findings, we propose that adults with higher educational qualifications who have relatively mild dyslexic difficulties may see themselves as more handicapped in the workplace than their peers and hence be more likely to self-report as dyslexic than a similarly affected person in a manual job.

In summary, a protocol such as the one described here provides a useful tool for screening for dyslexia and attention difficulties in adults. The finding that people who self-report as dyslexic rate themselves as having more difficulty with word finding, attention and hyperactivity than those who do not, underlines the fact that parents confer multiple continuous risks for learning disorders on their children. It is important for research following children at family risk of dyslexia to be aware of these effects.

## Appendix A: Adult Reading Questionnaire (ARQ): Items and Key to Scoring Responses

**Table:**

Question	Responses and Scores				
ARQ1 Do you think you are a good reader?	Yes	No	Don't Know		
	0	1	0.5		
ARQ 2 Can you read quickly and easily?	Yes	No	Don't know		
	0	1	0.5		
ARQ 3 How good is your spelling?	Good	Average	Poor	Very Poor	
	0	1	2	3	
ARQ 4 In your job, how often do you read?	Never	Rarely	Sometimes	Frequently	Always
	4	3	2	1	0
ARQ 5 Do you find it difficult to read words you haven't seen before (e.g., place names?)	Never	Rarely	Sometimes	Frequently	Always
	0	1	2	3	4
ARQ 6 Do you find it difficult to read aloud?	Never	Rarely	Sometimes	Frequently	Always
	0	1	2	3	4
ARQ 7 Do you find it difficult to find the right word to say?	Never	Rarely	Sometimes	Frequently	Always
	0	1	2	3	4
ARQ 8 Do you ever confuse the names of things?	Never	Rarely	Sometimes	Frequently	Always
	0	1	2	3	4
ARQ 9 Do you confuse left and right?	Never	Rarely	Sometimes	Frequently	Always
	0	1	2	3	4
ARQ 10 Do you have problems with organization or time management?	Never	Rarely	Sometimes	Frequently	Always
	0	1	2	3	4
ARQ 11 How often do you write in everyday life?	Never	Rarely	Sometimes	Frequently	Always
	4	3	2	1	0
ARQ 12. Based on this, do you think you are dyslexic?	Yes	No	Maybe		
	1	0	0.5		
ARQ 13. How would you rate your difficulties?	No difficulties	Mild	Moderate	Severe	
	0	1	2	3	
ARQ 14. Has anyone ever raised any concerns about your reading?	Yes	No			
	1	0			
ARQ 15. Have you ever had a diagnosis of dyslexia?	Yes	No			
	1	0			

## ABBREVIATIONS

ARQ: Adult Reading Questionnaire
ASRS: Adult ADHD Self-Report Scale
ADHD: Attention Deficit Hyperactivity Disorder
SRD: self-report of dyslexia
